# Human-AI collaboration in large language model-assisted brain MRI differential diagnosis: a usability study

**DOI:** 10.1007/s00330-025-11484-6

**Published:** 2025-03-07

**Authors:** Su Hwan Kim, Jonas Wihl, Severin Schramm, Cornelius Berberich, Enrike Rosenkranz, Lena Schmitzer, Kerem Serguen, Christopher Klenk, Nicolas Lenhart, Claus Zimmer, Benedikt Wiestler, Dennis M. Hedderich

**Affiliations:** 1 Department of Diagnostic and Interventional Neuroradiology, Klinikum rechts der Isar, School of Medicine and Health, Technical University Munich, Munich, Germany; 2 Department of Diagnostic and Interventional Radiology, Klinikum rechts der Isar, School of Medicine and Health, Technical University Munich, Munich, Germany; 3 AI for Image-Guided Diagnosis and Therapy, Technical University of Munich, Munich, Germany

**Keywords:** Large language models, Artificial intelligence, Brain, Magnetic resonance imaging, Differential diagnosis

## Abstract

**Objectives:**

This study investigated the impact of human-large language model (LLM) collaboration on the accuracy and efficiency of brain MRI differential diagnosis.

**Materials and methods:**

In this retrospective study, forty brain MRI cases with a challenging but definitive diagnosis were randomized into two groups of twenty cases each. Six radiology residents with an average experience of 6.3 months in reading brain MRI exams evaluated one set of cases supported by conventional internet search (Conventional) and the other set utilizing an LLM-based search engine and hybrid chatbot. A cross-over design ensured that each case was examined with both workflows in equal frequency. For each case, readers were instructed to determine the three most likely differential diagnoses. LLM responses were analyzed by a panel of radiologists. Benefits and challenges in human-LLM interaction were derived from observations and participant feedback.

**Results:**

LLM-assisted brain MRI differential diagnosis yielded superior accuracy (70/114; 61.4% (LLM-assisted) vs 53/114; 46.5% (conventional) correct diagnoses, *p* = 0.033, chi-square test). No difference in interpretation time or level of confidence was observed. An analysis of LLM responses revealed that correct LLM suggestions translated into correct reader responses in 82.1% of cases (60/73). Inaccurate case descriptions by readers (9.2% of cases), LLM hallucinations (11.5% of cases), and insufficient contextualization of LLM responses were identified as challenges related to human-LLM interaction.

**Conclusion:**

Human-LLM collaboration has the potential to improve brain MRI differential diagnosis. Yet, several challenges must be addressed to ensure effective adoption and user acceptance.

**Key Points:**

***Question*** *While large language models (LLM) have the potential to support radiological differential diagnosis, the role of human-LLM collaboration in this context remains underexplored.*

***Findings*** *LLM-assisted brain MRI differential diagnosis yielded superior accuracy over conventional internet search. Inaccurate case descriptions, LLM hallucinations, and insufficient contextualization were identified as potential challenges.*

***Clinical relevance*** *Our results highlight the potential of an LLM-assisted workflow to increase diagnostic accuracy but underline the necessity to study collaborative efforts between humans and LLMs over LLMs in isolation.*

**Graphical Abstract:**

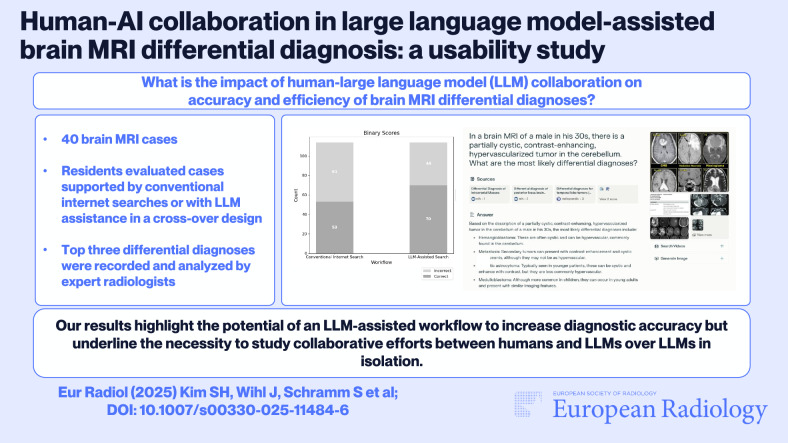

## Introduction

Radiological differential diagnosis plays a crucial role in clinical care, profoundly influencing diagnostic and therapeutic decisions. Accurate determination of relevant differential diagnoses from image findings demands highly specialized knowledge of anatomy and pathophysiology along with proficiency in recognizing visual patterns and synthesizing comprehensive clinical information.

Recent studies suggest the emerging potential of large language models (LLMs) to execute radiological differential diagnosis based on case presentations [1–6]. These studies compared the diagnostic suggestions of an LLM to expert assessments or confirmed diagnoses. In one recent study, radiologists aided by GPT-4 demonstrated slightly improved diagnostic performance and significantly higher confidence levels but reported hallucinations in 7.4% of responses [7]. Yet, the intricate interactions between human users and LLM systems in this context remain to be explored in more detail.

Previous literature reveals the critical impact of human-AI interaction on diagnostic performance in radiology [8–10]. One study employing an AI-based mammogram classification system demonstrated that inexperienced and experienced readers alike are susceptible to automation bias, which describes the inclination of human users to adhere to incorrect recommendations from automated decision-making systems [8]. Similarly, incorrect AI results were shown to negatively impact radiologist performance in lung cancer detection based on chest radiography [9]. Yet another study highlighted the significance of establishing effective human-AI collaboration protocols in AI-assisted knee MRI reading [10]. Analogously, elements of human-AI collaboration could affect the outcomes of LLM-assisted differential diagnosis. In real-world practice, it is plausible that radiologists or radiology residents would use LLMs as an adjunct tool to support diagnostic reasoning rather than for fully autonomous differential diagnosis [11]. Under these circumstances, the human medical professional assumes a pivotal position in contextualizing the available clinical and visual information, formulating the prompt, critically reviewing the LLM response, and conducting further research to eventually derive a conclusion. Especially considering the well-known propensity of LLM systems to generate factually incorrect information (so-called hallucinations) [12, 13], a comprehensive evaluation of how users realistically interact with these systems is imperative [7].

Therefore, this study aimed to investigate the impact of human-LLM collaboration on the accuracy and efficiency of brain MRI differential diagnosis.

## Methods

Written informed consent was waived by the Institutional Review Board. A preprint version of this article has been published previously [14].

### Study sample

**Fig. 1:**
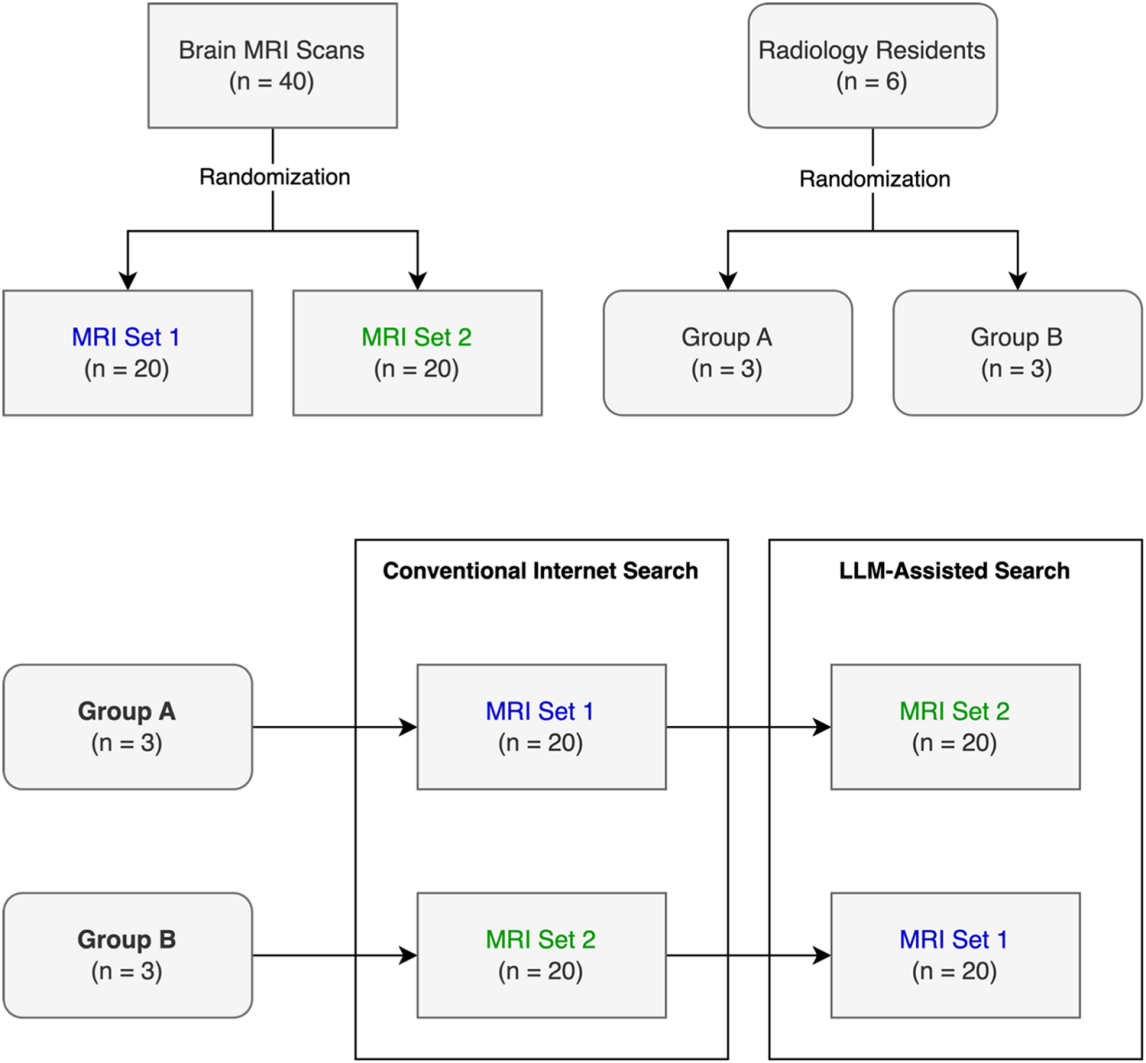
Study design. Forty brain MRI scans were randomized into two sets. Six radiology residents were randomized into two groups. Residents were instructed to solve brain MRI cases either using conventional internet search or an LLM system (one MRI set each)

In this retrospective study, six radiology residents with an average neuroradiology experience of 6.3 months at the time of the study participation (min: 2 months, max: 11 months) were recruited from the local departments of radiology and neuroradiology as participants of the usability study and randomized into two groups. A total of forty brain MRI exams obtained between 01/01/2016 and 12/31/2023 were selected from the local imaging database and randomized into two sets (Fig. 1, Supplement 1). In all forty cases, definitive diagnoses had been confirmed histopathologically (42.5%, 17/40) or by independent agreement of two neuroradiologists, taking into account all relevant clinical follow-up information (57.5%, 23/40). To clearly highlight the pathology the reading focused on, arrow annotations were added to each brain MRI scan by one radiologist with 3 years of neuroradiology experience (SS). In cases with multiple findings of the same type (e.g., multiple toxoplasmosis lesions), several annotations were made to indicate findings. In cases with several distinct findings, only one key finding was marked (e.g., only distended optic nerve sheaths were marked in a case of idiopathic intracranial hypertension). Selection criteria for participants and MRI scans are shown in Table 1.

**Table 1:** Inclusion criteria for brain MRI scans

Adult patient (at least 18 years old at the time of exam)	To exclude conditions highly specific to a pediatric subpopulation
Focal image finding	To define a clearly delimited finding for the participants to interpret
Confirmed diagnosis	Diagnosis confirmed histopathologically or by independent agreement of two neuroradiologists (if no histological specimen is available)
Initial diagnosis at the time of exam	To simulate a more realistic scenario requiring differential diagnosis
Non-trivial, less common conditions	To ensure cases were challenging enough to require further research

### LLM and Chatbot interface

PerplexityAI (© Perplexity AI Inc.; www.perplexity.ai) was chosen as the chatbot interface, given its ability to access real-time web content and to indicate its sources of information [15, 16]. GPT-4 (Generative Pre-trained Transformer 4), OpenAI’s most up-to-date LLM at the beginning of the study (November 2023), was selected as the model powering the search queries.

### Study design

Each participant was tasked to determine the three most likely differential diagnoses of the annotated image finding in the brain MRI scans via conventional internet search for half (*n* = 20) and via LLM-assisted search (© PerplexityAI) for the other half (*n* = 20) (Fig. 1). At the beginning of the LLM-assisted reading session, the LLM system was introduced to participants in a 10–15-min training session to ensure familiarity with its operation and functionality. During this training, participants explored the tool using a selection of three sample brain MRI cases not included in the test sample. For each of the three cases, a sample prompt including case details (age range, sex, symptoms, MRI findings) and an explicit instruction to name the most likely differential diagnoses was provided. However, participants were not restricted to a specific prompting strategy and were allowed to explore alternative approaches at will. Readers examined the cases over the course of four sessions, spanning from 3 November 2023 to 8 July 2024.

Cases were excluded from the analysis if participants were familiar with the case, the image finding was not recognized despite the annotation, or the correct diagnosis could be determined confidently without further research. Importantly, participants performing LLM-assisted searches were allowed to conduct additional conventional internet searches to validate LLM suggestions. Participants were allowed to submit up to three differential diagnoses, ranked by likelihood. Alongside the MRI scan, participants received demographic and clinical details of the patient’s case. All prompts were phrased in English. Interpretation times were measured using a time-tracking software (Toggl Track, © TogglOÜ). Level of confidence was recorded for each case on a 5-point Likert scale (1: very low confidence, 5: very high confidence). During the usability sessions, notes of relevant observations and comments were taken by S.H.K. and S.S. Before the first session, participants completed a brief survey collecting information on demographics, level of radiological experience, and prior exposure to LLMs. Following the usability testing, a follow-up questionnaire was administered to assess the LLM search engine in terms of clinical applicability, quality of its responses, ease of adoption, and overall experience.

### Analysis

To determine the accuracy of differential diagnoses, two different scoring systems were applied. In the first approach, participant responses were classified as “correct” if the correct diagnosis was included among the submitted differential diagnoses and “incorrect” if it was not (binary scoring system). In the second approach, participant responses were assigned a score from 0 to 3, depending on the rank of the correct diagnosis within the reader response (numeric scoring system). A score of 3 was assigned if the correct diagnosis was ranked first, 2 if ranked second, 1 if ranked third, and 0 if the correct diagnosis was not included. Inferential statistics were performed using the chi-square test for binary scores and the Mann–Whitney U test for numeric scores and level of confidence. Interpretation times were analyzed using the independent Student’s *t*-test. The level of significance was set at *p* < 0.05. Likert-scale questions of the questionnaires were analyzed by descriptive statistics. Cohen’s h was reported as a measure of effect size to assess the difference in the proportion of correct answers (binary score of 1) between the two groups. To account for possible variability introduced by readers and case difficulty, a complementary linear mixed-effects model was employed, with the binary score as dependent variable. Workflow (conventional vs LLM-assisted) was defined as the fixed effect, participant and case as random effects.

Logs of the LLM chatbot (PerplexityAI) were reviewed to quantify the number of performed queries as well as the number of differential diagnoses and internet sources indicated in the LLM responses. Queries were grouped by content (differential diagnosis, radiographic features, sample images, anatomy, other). Sources were categorized into journal articles and online platforms.

LLM logs were further screened for incorrect user inputs (e.g., inaccurate description of image findings) and hallucinations in LLM responses (information inconsistent with widely accepted radiological knowledge) by three radiologists (2, 3 and 8 years of experience in reading brain MRI exams). For this purpose, imaging characteristics of diagnoses indicated in LLM responses were compared to the respective descriptions on the online platform www.radiopaedia.org which is a validated source of radiological knowledge.

Based on the presence of the correct diagnosis in the LLM response, and the presence of the correct diagnosis in the final reader response (top 3 differential diagnoses), a contingency table was created to represent the extent to which correct LLM suggestions translated into correct diagnostic assessments of readers.

Qualitative data from observation notes, comments and questionnaire responses were summarized in tables, categorizing data by relevant topics. Data manipulation, analysis and visualization were performed using Python (version 3.9.7).

### Sample size calculation

While literature on the impact of LLM assistance on the diagnostic performance of radiology readers is very scarce, a small effect size of 0.2 was assumed based on related prior work [17]. As a result, adopting a statistical power of 80%, an α error probability of 0.05, and a two-tailed Wilcoxon signed-rank test, a minimum sample size of 208 was determined (G*Power, v3.1).

## Results

A total of 12 out of 240 responses (6 responses in the conventional and LLM-assisted group each) were excluded from the analysis due to the participant’s familiarity with the MRI scan (*n* = 1), their inability to recognize the annotated image finding (*n* = 2), or their ability to confidently determine the correct diagnosis without further research (*n* = 9).

### Quantitative findings

**Fig. 2:**
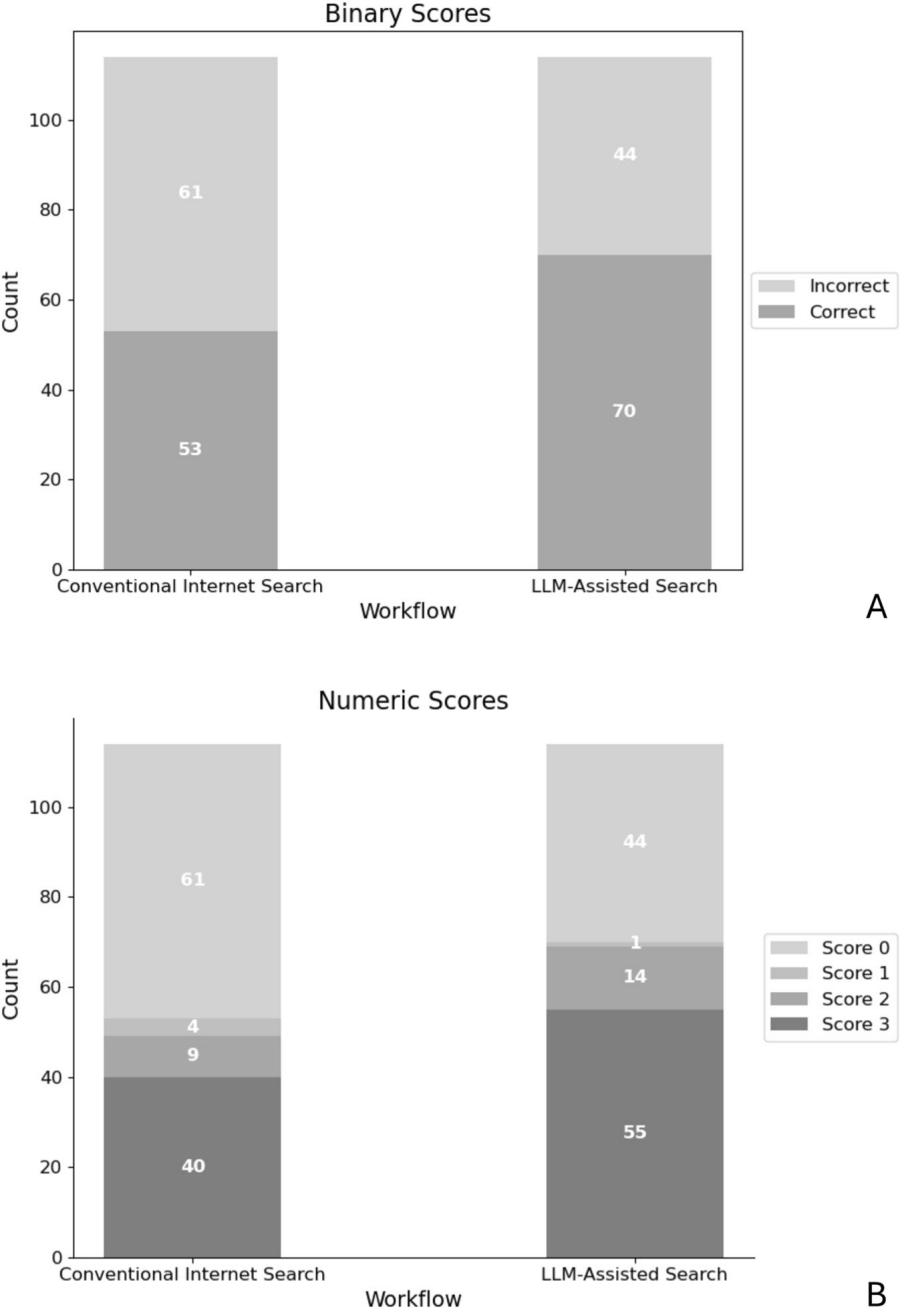
Accuracy of differential diagnoses by workflow. **A** Binary scoring system. Participant responses were classified as either correct or incorrect. LLM-assisted workflow yielded superior scores (*p* = 0.033). **B** Numeric scoring system. A participant’s response was assigned a score between 0 and 3, depending on the rank of the correct diagnosis within the response (3: correct diagnosis ranked first, 0: correct diagnosis not included in response). LLM-assisted workflow yielded superior scores (*p* = 0.021)

**Fig. 3:**
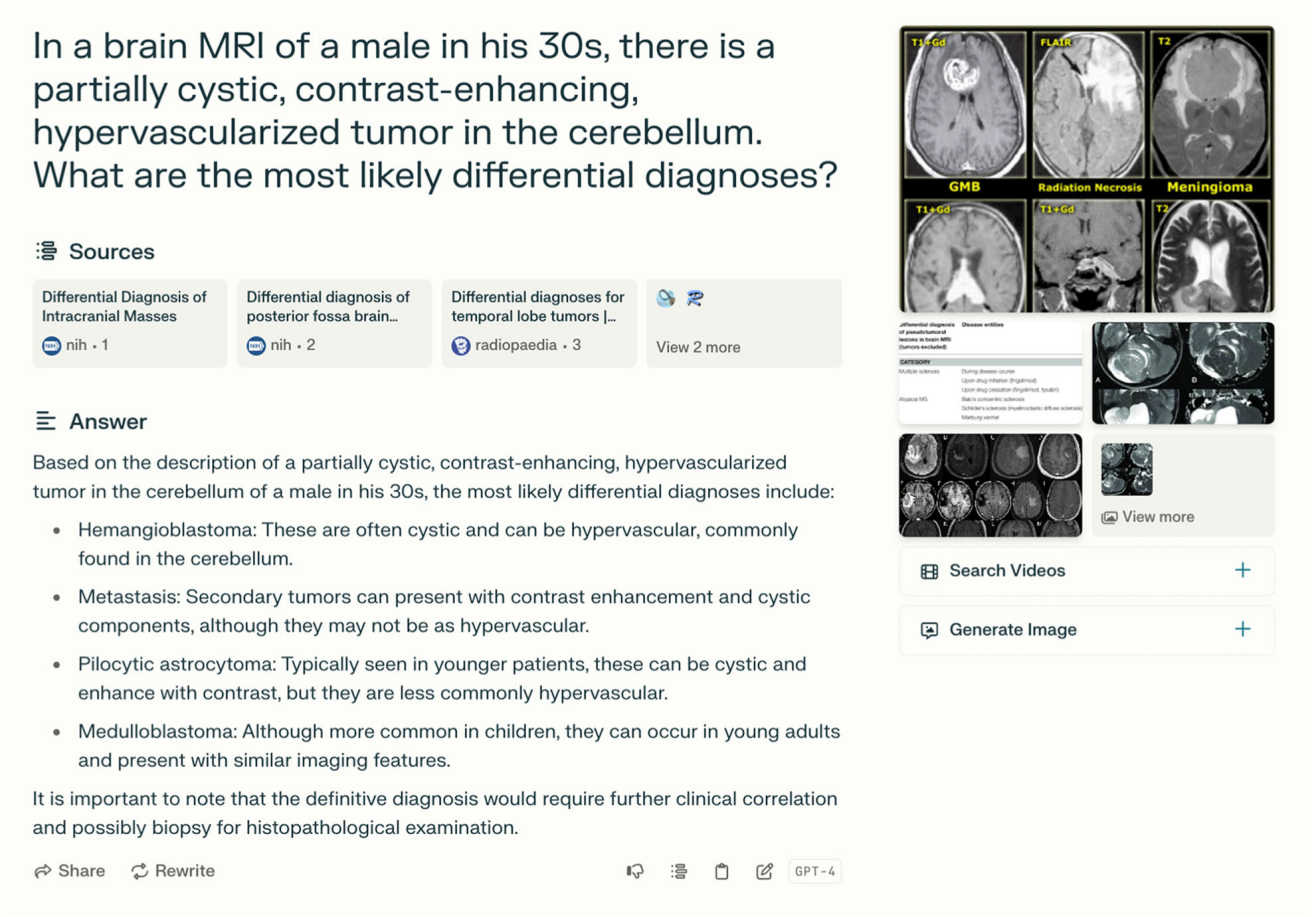
Screenshot of a sample LLM query (PerplexityAI). The correct diagnosis sought in this case was hemangioblastoma

LLM-assisted brain MRI differential diagnosis yielded superior accuracy, as evaluated by both binary (70/114; 61.4% (LLM-assisted) vs 53/114; 46.5% (conventional) correct diagnoses, *p* = 0.033) and numeric scoring approach (median score of 1 (LLM-assisted) vs 0 (conventional), *p* = 0.021) (Fig. 2). The linear mixed-effects model confirms an association of the LLM-assisted workflow with significantly higher binary scores (β = 0.150, SE = 0.065, *z* = 2.309, *p* = 0.021). Based on the binary scores, a Cohen’s h of 0.30 was determined, indicating a small to medium effect size. No difference in interpretation time (7.38 ± 3.35 min (LLM-assisted) vs 7.17 ± 3.49 min (conventional), *p* = 0.64) was observed. Similarly, the level of confidence did not differ significantly (median of 3 (LLM-assisted) vs 3 (conventional), *p* = 0.50). A screenshot of a sample LLM response is shown in Fig. 3.

### LLM response evaluation

113 out of 114 cases reviewed with LLM assistance were analyzed. In one case, the respective logs could not be retrieved due to technical issues of PerplexityAI. The mean number of LLM queries per case amounted to 2.12. The majority of queries directly pertained to possible differential diagnoses (64.2%), followed by queries related to sample images (11.1%) and image characteristics of suspected pathologies (3.7%). The LLM tool referenced an average of 13.0 internet sources per response. 72.0% of the referenced internet sources were journal articles (Supplement 2). Per case, a mean of 7.59 differential diagnoses were indicated in LLM responses. An analysis of the LLM prompts provided by readers revealed incorrect or inaccurate information in 9.2% of cases (= 4.3% of queries). LLM outputs included hallucinatory statements in 11.5% of cases (= 5.4% of queries) (Table 2). Nine out of 13 hallucinatory statements were classified as incorrect descriptions of imaging characteristics, whereas 4 out of 13 were related to incoherent associations of anatomical locations (Supplement 3).

**Table 2:** LLM response metrics

Metric	Value (± SD)
Mean number of queries per case	2.12 ± 1.56
Mean number of DDx indicated per case	7.59 ± 5.23
Mean number of internet sources indicated in LLM response	13.0 ± 12.34
Relative frequency of incorrect LLM inputs/prompts (per case)	9.2%
Relative frequency of incorrect LLM inputs/prompts (per query)	4.3%
Relative frequency of hallucinations (per case)	11.5%
Relative frequency of hallucinations (per query)	5.4%

*SD* standard deviation

In 60 out of 73 cases where the LLM response included the correct diagnosis, readers identified the correct diagnosis (82.1%). Inversely, readers overlooked the correct diagnosis indicated in the LLM response in 13 out of 73 cases (17.9%). In 10 out of 70 cases where readers indicated the correct diagnosis, the correct diagnosis was not included in the LLM response (14.3%), suggesting that the answer was found through additional internet research (Table 3).

**Table 3:** Contingency table

	Correct diagnosis NOT in LLM response	Correct diagnosis in LLM response	Total
Correct diagnosis NOT in reader response	30 (LLM did not suggest correct diagnosis, and reader did not find correct diagnosis through additional research either)	13 (reader did not find correct diagnosis despite correct LLM suggestion)	43 (38.1%)
Correct diagnosis in reader response	10 (reader found correct diagnosis through additional internet research)	60 (LLM helped reader find the correct diagnosis)	70 (62.0%)
Total	40 (35.4%)	73 (64.6%)	113 (100.0%)

### Reader feedback and observations

**Fig. 4:**
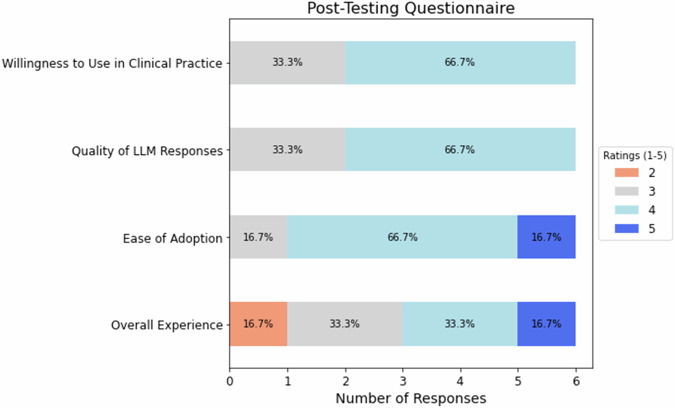
Post-testing questionnaire results (1: very low, 5: very high)

Questionnaire results revealed a moderately positive evaluation of the LLM-assisted workflow. Participants showed a slight tendency in favor of using the LLM tool in clinical practice (median: 4). Quality of LLM responses was rated rather positively (median: 4). The LLM system was easily adopted into the diagnostic workflow by most participants (median: 4). The overall experience of the LLM-assisted search workflow was mixed (median: 3.5) (Fig. 4).

Several challenges in human-LLM interaction related to both human errors and technical limitations were observed (Table 4). Misleading or inaccurate search results attributable to human errors included inaccurate descriptions of image findings (e.g., describing the location of a cerebral cavernous venous malformation as “extra-axial”) or an omission of relevant imaging features (e.g., omission of multinodular morphology of a multinodular and vacuolating neuronal tumor; MVNT). LLM search, in contrast, exhibited bias based on clinical information irrelevant to the diagnosis (e.g., history of kidney transplantation in a patient with posterior reversible encephalopathy syndrome; PRES) or connotative terminology (e.g., the term “juxtacortical” was strongly associated with multiple sclerosis). Participant feedback on LLM-assisted differential diagnosis is illustrated in Table 5. Prior to the usability sessions, several participants expressed concerns about excessive reliance on the LLM system and consecutive impairment of their own radiological training. Following the reading sessions, the possibility to give flexible instructions to the LLM search engine regarding scope (e.g., quantity of differential diagnoses) and format (e.g., bullet points, table, sample images) of the search result was pointed out as a key advantage over conventional internet search. Participants believed that the usability of the LLM system for differential diagnosis could be enhanced by enabling voice-based interactions and improving the accuracy of returned sample images.

**Table 4:** Challenges during human-LLM interaction derived from observations and participant comments

Challenges	Examples
Omission of relevant clinical information	-Omitting the age of a patient with spinocerebellar ataxia (resulted in misleading suggestions such as atypical Parkinson syndrome)
Inclusion of clinical information irrelevant to the diagnosis	-Indicating recent history of head trauma in a patient with an incidental finding (benign enhancing foramen magnum lesion) -Indicating a history of kidney transplantation in a patient with posterior reversible encephalopathy syndrome (PRES)
Inaccurate description of imaging findings	-Describing the location of a cerebral cavernous venous malformation as “subarachnoid” or “extra-axial” -Searching for differential diagnoses of global brain atrophy in a patient with focal atrophy of the caudate head
Omission of relevant imaging features	-Omitting the bubbly or multinodular appearance of a multinodular and vacuolating neuronal tumor (MVNT) resulted in highly unspecific suggestions
Usage of connotative terminology	-Describing the location of a lesion as “juxtacortical” in a patient with MVNT (was strongly associated with multiple sclerosis)
LLM returning completely unrelated content (very few)	-Upon a request for sample images of progressive multifocal leukoencephalopathy (PML), images of the prime minister of India were returned

**Table 5:** Participant comments on LLM-assisted differential diagnosis

Theme	Comments
Concerns (pre-testing)	Residents might develop a strong dependence on the tool, educational effect could be impaired
	Excessive reliance on LLM tool can promote intellectual laziness and carelessness in image interpretation
	LLMs are known to create hallucinations, source of information is not always clear
	The role of the radiologist might become obsolete as core tasks are increasingly overtaken by AI tools including LLMs
Benefits	Possibility to phrase open questions rather than rigid keyword searches is liberating
	Links to information sources are embedded in the response
	Instructions can be given regarding the scope (e.g., quantity of differential diagnoses suggested) and format of the response (e.g., table, bullet points)
	The context of prior queries is retained so that follow-up questions can be posed
	Useful to obtain a quick overview of a broad range of differential diagnoses
Suggestions for improvement	Interaction with the LLM system via voice
	Improved search of relevant sample images
	Quantitative data regarding the probability of certain diagnoses in the presence of specific imaging features

## Discussion

In this study, we conducted usability experiments to explore the role of human-LLM collaboration in brain MRI differential diagnosis. Our results suggest that an LLM-assisted workflow has the potential to increase diagnostic accuracy as compared to conventional internet search. Yet, no clear effect on interpretation times and reader confidence was observed.

These results contrast a recent study by Siepmann et al that reported only minor, non-significant improvements in diagnostic performance but significantly higher confidence levels of readers with GPT-4 assistance [7]. These differences could be explained by the experience levels of readers, which were substantially lower in the current study, and support our hypothesis that inexperienced readers benefit more from LLM-assisted diagnosis than experienced ones. The rate of hallucinatory LLM responses in this study (5.4%) was only marginally lower than the one reported by Siepmann et al (7.4%). This is noteworthy, as it suggests that even an LLM search engine utilizing a retrieval-augmented generation method to support its responses with internet sources can return factually incorrect and misleading statements. Our finding that an average of 13.0 sources were cited per case further underscores the infeasibility for readers to verify every reference individually.

Our evaluation of LLM responses reveals deeper insights into the challenges of human-LLM interaction in radiological diagnosis. A post-hoc assessment of reader-generated LLM prompts showed inaccurate information in 9.2% of cases. This metric did not include omissions of relevant case details or imaging characteristics. In 17.8% of cases where the LLM response suggested the correct diagnosis, readers submitted an incorrect answer, highlighting the critical role of readers to prioritize relevant LLM suggestions and to validate them through complementary internet research. Readers typically correlated image findings with sample images of suggested diagnoses. Focused internet searches on trusted websites (e.g., Radiopaedia) proved more effective for this purpose, indicating that the joint use of LLMs and conventional internet search might outperform the exclusive use of LLMs.

**Fig. 5:**
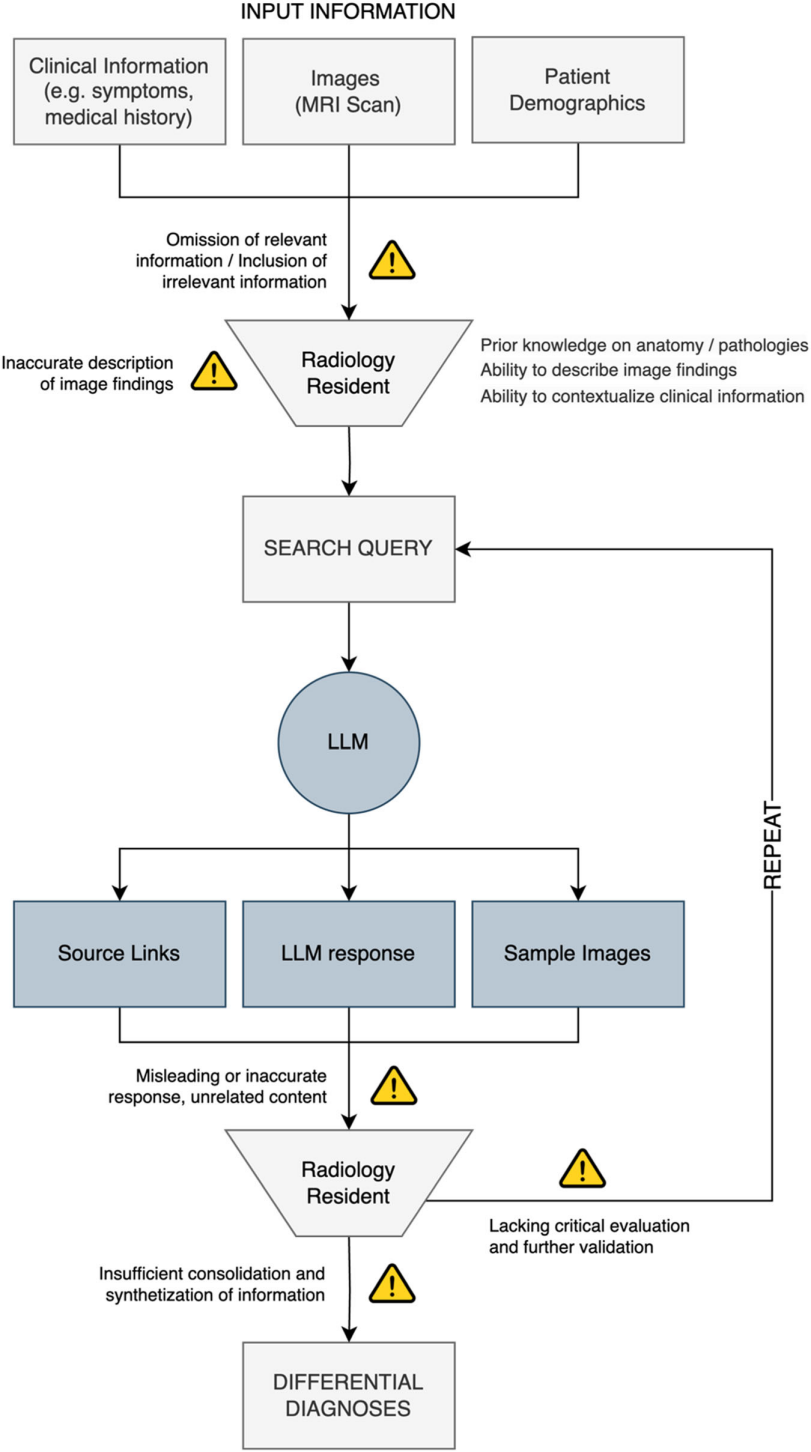
Conceptual model of human-LLM interaction for radiological differential diagnosis. Warning icons indicate potential sources of error in human-LLM interaction

A conceptual model illustrating the role of the human agent in LLM-assisted differential diagnosis and potential sources of error is shown in Fig. 5. Altogether, our results underline the necessity to study collaborative efforts between humans and LLMs over LLMs in isolation to better reflect real-world conditions. While LLMs can augment human capabilities, traditional neuroradiological expertise remains indispensable for their effective utilization.

Notably, rapid advancements in LLM technology demand continuous evaluations. Recent innovations, such as voice assistants enabling conversational interactions (e.g., as featured in OpenAI’s ChatGPT), are transforming LLM usability. Earlier, the introduction of speech recognition dictation systems enhanced productivity in radiology departments [18]. It is yet to be determined whether voice interaction with LLMs can yield similar benefits. The emergence of multimodal LLMs capable of processing image inputs is expected to create new possibilities. Few recent studies have demonstrated their potential to generate chest x-ray reports [19] and answer USMLE questions involving radiological images [20]. In radiological differential diagnosis, direct image processing by LLMs could significantly alter human-LLM interactions by eliminating the need for human image descriptions. Seamless integration of LLMs into established health information systems could further boost productivity. For instance, prompt generation could be streamlined by directly importing clinical information from a RIS or selecting a key image from a PACS system as input.

This study has several limitations. First, given that only radiology residents with little experience in neuroradiology were included, our findings might not directly apply to more experienced readers. Second, human-LLM interactions were evaluated in a controlled environment but not in a real-world clinical setting, where frequent interruptions and intense workload might alter reader behavior. Third, diagnostic performance of both readers and LLMs needs to be viewed in the context of inherent limitations of radiological diagnosis. We defined the gold standard diagnosis either histopathologically or based on neuroradiologist consensus incorporating all available follow-up information. Yet, in some cases even highly skilled radiologists are not able to determine the accurate pathology from imaging findings alone. Therefore, discrepancies between imaging-based differential diagnoses and true diagnoses may, in part, be attributed to these fundamental diagnostic challenges.

Fourth, the participants’ familiarity with the LLM system was limited, compared to their extensive experience with conventional internet search. Additional exposure and training could help users formulate more effective prompts, thereby reducing frustration and inefficiencies. Fifth, the LLM-based search engine (PerplexityAI) differs from other LLM-based tools in that it combines traditional search pipelines with LLMs, but potential limitations of this technical approach remain to be explored. Finally, a general-purpose LLM (GPT-4) was used. Models specifically trained for medicine (e.g., MED-PALM, GlassAI) [21**–**23] or radiology [24**–**26] are anticipated to further enhance clinical utility.

In conclusion, our study shows that human-LLM collaboration has the potential to enhance differential diagnosis of brain MRI but recognizes the necessity to mitigate both human and technical errors to maximize effectiveness.

## Supplementary information

ELECTRONIC SUPPLEMENTARY MATERIAL

## Abbreviations

LLM: Large language model
MRI: Magnetic resonance imaging
