# Supplementary material for: Human-AI collaboration in large language model-assisted brain MRI differential diagnosis: a usability study
Source: Eur Radiol. 2025 Mar 7;35(9):5252–63. doi: 10.1007/s00330-025-11484-6 (PMC12350562; doi:10.1007/s00330-025-11484-6)
Supplement: Supplementary file 1 — ELECTRONIC SUPPLEMENTARY MATERIAL [file 330_2025_11484_MOESM1_ESM.pdf]

Human-AI Collaboration in Large Language Model-Assisted Brain MRI Differential Diagnosis: A Usability Study

ELECTRONIC SUPPLEMENTARY MATERIAL

Eur Radiol (2025) Kim SH, Wihl J, Schramm S, et al.

Supplement 1: Brain MRI case overview. Clinical information were obtained from radiology request forms.

ID	Age Range	Sex	Clinical Information	Correct Diagnosis
**A01**	36 - 40	F	Intracranial mass in external CT scan, headache	Midline glioma
**A02**	56 - 60	F	Confusion, immunosuppression post-kidney transplant	Posterior reversible encephalopathy syndrome (PRES)
**A03**	31 - 35	M	Dizziness, headache	Hemangioblastoma
**A04**	36 - 39	M	Dysarthria, dysphagia, psychomotor slowing, suspected HIV infection, intracerebral lesions	Toxoplasmosis
**A05**	41 - 45	F	Suspected brain abscess, patent foramen ovale	Echinococcosis
**A06**	26 - 30	M	Headache and papilledema	Central neurocytoma
**A07**	36 - 40	F	Sensory-motor hemiparesis left	Tumefactive multiple sclerosis (MS)
**A08**	41 - 45	F	Progressive gait ataxia, upper limb ataxia for 2 years	Spinocerebellar ataxia
**A09**	56 - 60	M	Intracranial mass lesion in external images	Subependymoma
**A10**	56 - 60	M	Organic psychosyndrome for 3 months, psychomotor retardation, dysarthria	Limbic encephalitis
**A11**	41 - 45	F	Subacute onset of dizziness, ataxia and apraxia since a few days, improvement of symptoms under treatment with cortison. Sensorineural hearing loss of low frequencies.	Susac syndrome
**A12**	51 - 55	M	Severe gait ataxia, occasional double vision, history of chronic alcohol abuse	Wernicke encephalopathy
**A13**	40-45	M	Known epilepsy. Now demonstrates with new psychosis. Patient is somnolent after administration of Lorazepam.	Heterotopia
**A14**	56 - 60	F	Acute desorientation	Cerebral venous thrombosis
**A15**	41 - 45	M	Incidental clival finding in external imaging, patient is asymptomatic	Ecchordosis physaliphora
**A16**	76 - 80	M	Suspected vascula dementia. Status post left carotid TEA.	État criblé
**A17**	56 - 60	M	Progressive memory disorder, mild tetraspasticity, gait disorder. History of chronic arterial hypertension.	Subcortical arteriosclerotic encephalopathy (M. Binswanger)
**A18**	76-80	M	Subacute severe left occipital headache with gait disorder and inclination for falling to the left. Acute hypertension with >200 mmHg systolic.	dAVF
**A19**	41 - 45	F	Flickering vision in the right eye, recurrent headaches, history of migraine. Suspicion of congestive papilledema on the right eye in ophthalmologic examination.	Idiopathic intracranial hypertension

**A20**	41 - 45	F	Psychotic episode with paranoid delusions. No history of prior psychiatric diseases.	Pineocytoma
**B01**	21 - 25	F	Ski injury with head impact, retrograde amnesia, initial confusion, subsequent improvement, MRI preferred due to young age	Benign enhancing foramen magnum lesion
**B02**	51 - 55	F	Newly diagnosed left sixth nerve palsy with diplopia, severe headaches for 2 days, left facial numbness for several weeks	Chondrosarcoma
**B03**	56 - 60	F	Repeated orofacial dyskinesias, parathymic behavior. Generalized brain atrophy in CT. DDx neurodegenerative disease	Huntington's disease
**B04**	36 - 40	M	Gait ataxia, dysarthria, depression	Creutzfeldt Jakob disease
**B05**	21 - 25	F	Left hemiparesis	Moya Moya disease
**B06**	36 - 40	M	Incidental finding in left temporal lobe in external imaging	Cerebral cavernous venous malformation
**B07**	31 - 35	F	Headache, history of Cesarean section and HELLP syndrome 3 weeks ago, HSV pneumonia	Cytotoxic lesions of the corpus callosum (CLOCC)
**B08**	46 - 50	M	Subacute ptosis, right oculomotor palsy, diplopia	Multinodular and vacuolating neuronal tumor (MVNT)
**B09**	31 - 35	M	Scalp lesion DDx lipoma DDx atheroma, Pt. noticed swelling a few months ago, has grown since	Sarcoma
**B10**	61 - 65	M	Severe left sensorineural hearing loss	Superficial siderosis
**B11**	41 – 45	F	Hearing loss in the left ear, occasional otorrhea, no vertigo	Choleasteatoma
**B12**	31 - 35	F	Status post traumatic brain injury. CT imaging revealed incidental mass located in the frontal horn of the left lateral ventricle.	SEGA (subependymal giant cell astrocytoma) / Tuberous sclerosis
**B13**	76 - 80	F	Acoustic hallucinations since several years. No speech disorder.	Mixed neuronal-glial tumor
**B14**	81 - 85	F	Rapidly progressive cognitive disorder	Frontotemporal dementia
**B15**	16 - 20	F	Patient with history of bipolar disorder, incidental lesion in 3rd ventricle<br>Occasional headache, no dizziness or vomiting	Colloid cyst
**B16**	61 - 65	F	Progressive right-sided hemiparesis, history of chronic lymphocytic leukemia	Progressive multifocal encephalopathy (PML)
**B17**	46 - 50	F	Proptosis of the left eye and visual impairment and double vision	Cavernous hemangioma of the orbit
**B18**	81 - 85	F	Progressive facial pain, elevated inflammation markers, right abducens nerve palsy	Osteomyelitis of the skull base
**B19**	46 - 50	M	History of trigeminal neuralgia for 9 months. Dentis carried out two root canal treatments without any significant improvement of symptoms.	Epidermoid cyst
**B20**	21 - 25	F	Recurrent numbness in the left arm, dizziness	Focal cortical dysplasia

Supplement 2: Classification of LLM queries and LLM source references.

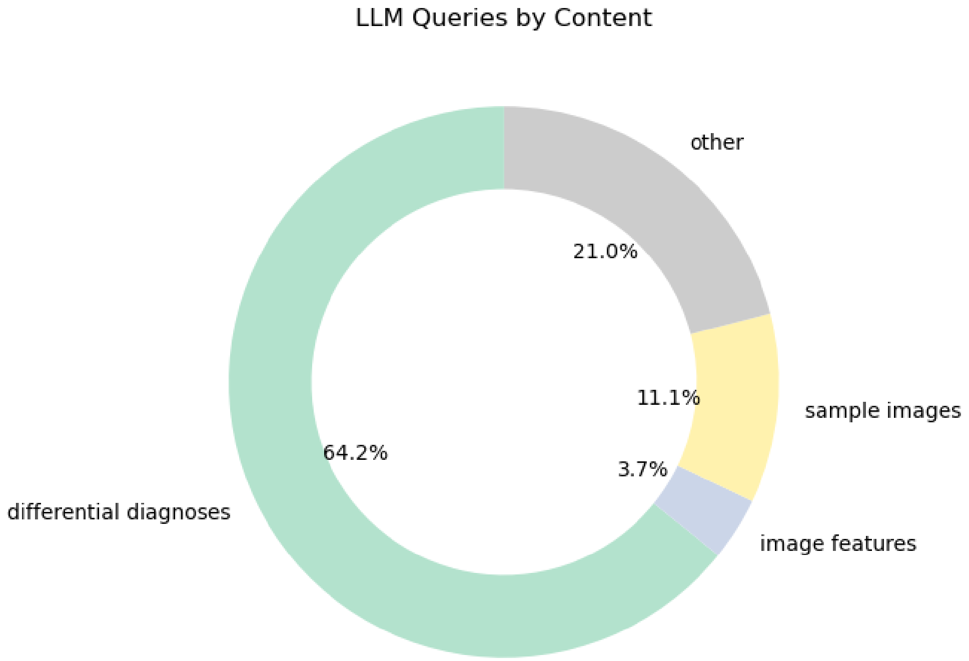

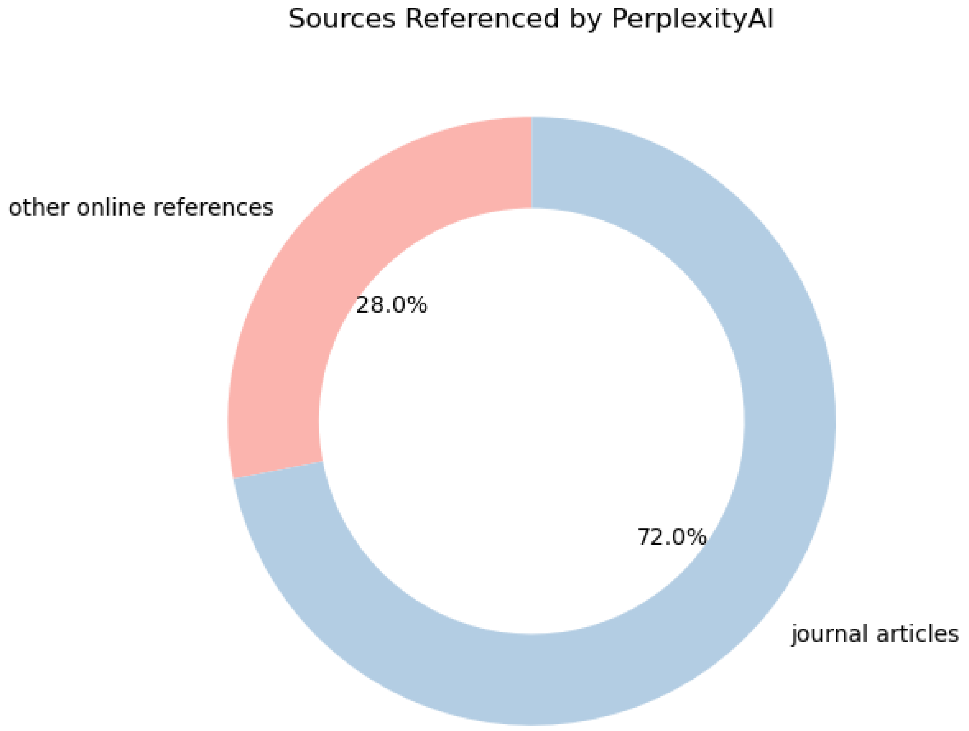

Supplement 3: Hallucinatory LLM responses.			
**Correct Diagnosis**	Input	LLM Response	Explanation
**Epidermoid cyst**	Give me 3 differentials for a mass growing around the brainstem with mass effect (the mass is FLAIR hyperintense, shows no enhancement, and has a part in the inner ear canal).	Hypertrophic olivary degeneration: This is a rare condition where disruption of the dentato-rubro-olivary pathway leads to hypertrophy and T2/FLAIR hyperintensity of the inferior olivary nucleus in the brainstem. It can mimic a mass lesion but typically does not enhance or extend into the inner ear canal.<br><br>Hallucination type: imaging features	The description of a "mass growing around the brainstem" doesn't fit the imaging characteristics of a hypertrophic olivary degeneration, which is confined to the olive.
**dAVF**	Male patient in his late 70s presenting with subacute severe left occipital headache with gait disorder and inclination for falling to the left. In addition, he shows acute hypertension with >200 mmHg. The brain MRI shows signal loss on T2/FLAIR weighted images yet contrast-enhancing occipital veins. What are the 5 most likely differential diagnoses? Provide an explanation for each differential diagnosis.	Cerebral venous thrombosis (CVT)<br>CVT is a strong possibility given the severe occipital headache, gait disturbance, and MRI findings of contrast-enhancing occipital veins. The signal loss on T2/FLAIR could represent thrombosed veins. CVT can cause localized edema and venous infarction, leading to neurological deficits like gait disorders.<br><br>Hallucination type: imaging features	T2/FLAIR signal loss of veins represents normal flow void and does not indicate thrombosis.
**Epidermoid cyst**	A 45-year-old male has a history of trigeminal neuralgia for 9 months. On MRI there is a T2w inhomogeneous mass perimesencephalic and extraparenchymal without contrast enhancement. Give me the most common differential diagnosis in bullet points.	Demyelinating lesion (e.g., multiple sclerosis plaque)<br><br>Hallucination type: location	Even though the lesion was described as "extraparenchymal", an intraparenchymal lesion ("demyelinating lesion") was suggested
**Susac syndrome**	A 40-year-old female presents with subacute dizziness, ataxia, and apraxia for a few days. The symptoms improve under treatment with steroids. She also has hearing loss at low frequencies. The MRI shows small T2-hyperintensities in the corpus callosum and the pons. Name the most probable differential diagnoses with an explanation for each.	Cerebellar Ataxia:<br>Reasoning: The patient's symptoms of ataxia and dizziness are characteristic of cerebellar ataxia. The MRI findings of small T2-hyperintensities in the corpus callosum and pons could indicate cerebellar involvement […].<br><br>Hallucination type: location	T2-hyperintensities in the corpus callosum and pons do NOT indicate cerebellar involvement

**Wernicke encephalopathy**	A 50-year-old male patient presents with severe gait ataxia and occasional double vision. He has a history of chronic alcohol consumption. The MRI presents the finding of contrast enhancement in the mammillary bodies. Name the most probable differential diagnoses with an explanation.	Miller-Fisher Syndrome (MFS):<br>Explanation: MFS is a rare autoimmune disorder that can cause gait ataxia, double vision, and other neurological symptoms. Although it is less likely to be directly related to chronic alcohol consumption, the symptoms and MRI findings are consistent with MFS [...].	Contrast enhancement of the mamillary bodies is not a typical feature of Miller-Fisher syndrome
**Chondrosarcoma**	Mass surrounding the carotid artery, contrast-enhancing and FLAIR hyperintense, reaching up into the temporal lobe. What are the differentials? Present some pictures of the named differentials.	Hallucinations:<br><br>Cerebral Autosomal Dominant Arteriopathy with Subcortical Infarcts and Leukoencephalopathy (CADASIL): This is a genetic disorder that causes stroke and other impairments [...].<br><br>Exostosis: This is a benign outgrowth of bone, which can cause mass effect.<br><br>Hallucination type: imaging features	The description of a mass surrounding the ICA does not fit CADASIL<br><br>The FLAIR hyperintensity and contrast enhancement does not fit exostosis
**Creutzfeldt Jakob disease**	A 37-year-old male, symmetric diffusion restriction in the nucleus caudatus, putamen, and bilateral thalamus in brain MRI. What are the differentials?	Huntington's Disease (HD): This is a neurodegenerative disorder characterized by the progressive loss of neuronal cells in the basal ganglia, particularly the caudate nucleus and putamen. The characteristic imaging finding of HD is bilateral striatal (caudate nucleus and putamen) atrophy.<br>[...]<br>Parkinson's Disease: The caudate nucleus and putamen are implicated in Parkinson's disease […].<br><br>Hallucination type: imaging features	Bilateral diffusion restriction of the nucleus caudatus, putamen and thalamus is not consistent with Huntington's disease or Parkinson's disease
**Cytotoxic lesions of the corpus callosum (CLOCC)**	A 35-year-old female, brain MRI shows a diffusion restriction in the splenium, no contrast enhancement. Provide differential diagnosis and some images.	Acute Hippocampal Disorders: These can show hyperintensity on DWI and T2-weighted images. The lesions may show reversible restricted diffusion. However, these typically involve the hippocampus rather than the splenium.<br><br>Hallucination type: location	Hippocampal disorders are suggested as potential differential diagnoses for a corpus callosum lesion.
**Subependymoma**	Please provide the differential diagnosis for a FLAIR centrally hypointense and peripherally	Multiple Sclerosis (MS): MS can present with lesions that are hypointense centrally and	Several intraparenchymal pathologies (multiple sclerosis, cerebral infarction) are

	hyperintense intraventricular non-contrast-enhancing lesion on MRI in a 59-year-old male.	hyperintense peripherally on T2-FLAIR sequences [...]<br><br>Toxic or Metabolic Brain Disorders: Certain toxic or metabolic disorders can present with hyperintense lesions on T2-weighted and FLAIR images [...].<br><br>Cerebral Infarction: Infarcts can appear hyperintense on FLAIR images [...].<br><br>Hallucination type: location	mentioned as differential diagnoses for an intraventricular lesion.
**Hemangioblastoma**	What are the differential diagnoses for a cystic lesion in the cerebellum with gadolinium enhancement?	Arachnoid Cyst: Although less common in the cerebellum, arachnoid cysts can sometimes show gadolinium enhancement, especially if there is inflammation or haemorrhage associated with the cyst.<br><br>Hallucination type: imaging features	Arachnoid cysts do not exhibit contrast enhancement (the indicated source does not mention this either).
**Limbic encephalitis**	A patient with psychiatric changes. Brain MRI shows a T2 hyperintensity of the limbic system. What are the possible diagnoses?	Alzheimer's Disease: Early stages of Alzheimer's disease can present with psychiatric symptoms and may show T2 hyperintensity in the limbic system on MRI.<br><br>Hallucination type: imaging features	T2 hyperintensity of the limbic system is not a typical feature of Alzheimer's disease. Also, this feature was not mentioned in the indicated source.
**Creutzfeldt Jakob disease**	I have an MRI of a 37-year-old female with symptoms of ataxia, dysarthria, and depression. There is a diffusion restriction in both the nucleus caudatus and both putamen. What are the most common differential diagnoses? Give me bullet points.	Huntington's disease: This is a progressive brain disorder that causes uncontrolled movements, emotional problems, and loss of thinking ability. The caudate nucleus and putamen are typically affected in this condition.<br><br>Hallucination type: imaging features	Bilateral diffusion restriction of the nucleus caudatus and putamen is not a imaging feature of Huntington's disease
**Superficial siderosis**	What are the differential diagnoses of hypointensity in T1 and FLAIR of the cerebellar folia?	Acute cerebellitis: This can cause cortical hypointensity on T1 and FLAIR images<br><br>Hallucination type: imaging features	Acute cerebellities typically shows T2/FLAIR hyperintensities (as also described in the cited source)
